# A murine glaucoma model induced by rapid *in vivo* photopolymerization of hyaluronic acid glycidyl methacrylate

**DOI:** 10.1371/journal.pone.0196529

**Published:** 2018-06-27

**Authors:** Chenying Guo, Xin Qu, Nalini Rangaswamy, Barrett Leehy, Chuanxi Xiang, Dennis Rice, Ganesh Prasanna

**Affiliations:** Department of Ophthalmology, Novartis Institutes for BioMedical Research, Cambridge, MA, United States of America; Schepens Eye Research Institute, Massachusetts Eye & Ear, Harvard Medical School, UNITED STATES

## Abstract

Glaucoma is an optic neuropathy commonly associated with elevated intraocular pressure (IOP) resulting in progressive loss of retinal ganglion cells (RGCs) and optic nerve degeneration, leading to blindness. New therapeutic approaches that better preserve the visual field by promoting survival and health of RGCs are highly needed since RGC death occurs despite good IOP control in glaucoma patients. We have developed a novel approach to reliably induce chronic IOP elevation in mouse using a photopolymerizable biomatrix, hyaluronic acid glycidyl methacrylate. This is achieved by rapid *in vivo* crosslinking of the biomatrix at the iridocorneal angle by a flash of ultraviolet A (UVA) light to impede the aqueous outflow pathway with a controllable manner. Sustained IOP elevation was induced after a single manipulation and was maintained at ~45% above baseline for >4 weeks. Significant thinning of the inner retina and ~35% reduction in RGCs and axons was noted within one month of IOP elevation. Optic nerve degeneration showed positive correlation with cumulative IOP elevation. Activation of astrocytes and microglia appeared to be an early event in response to IOP elevation preceding detectable RGC and axon loss. Attenuated glial reactivity was noted at later stage where significant RGC/axon loss had occurred suggesting astrocytes and microglia may play different roles over the course of glaucomatous degeneration. This novel murine glaucoma model is reproducible and displays cellular changes that recapitulate several pathophysiological features of glaucoma.

## Introduction

Glaucoma is the leading cause of irreversible blindness loss worldwide, it has been estimated that one in 40 adults over the age of 40 suffers from glaucoma [1]. Glaucoma is a multifactorial central nervous system (CNS) neurodegenerative disease characterized by cupping of the optic disc, progressive loss of retinal ganglion cells (RGCs) and their axons in the optic nerve tract. Elevated IOP is one major risk factor for primary open angle glaucoma (POAG)–the most common form of glaucoma accounting for 74% of all types[2]; however glaucoma can develop in the presence of seemingly normal IOP [3–7]. Other risk factors for the development of glaucomatous neuropathy include age, race, inflammation, oxidative and metabolic stresses, blood flow disturbances and genetic background [8–10]. Despite the various causative and risk factors, the common final pathway for all types of glaucoma is the loss of RGCs and optic nerve degeneration accompanied by cupping of the optic disc.

The current clinical standard of care for glaucoma is IOP lowering therapy through pharmaceutical or surgical approaches; however IOP reduction is not sufficient to stop progressive visual field loss in patients even when IOP is adequately controlled [3–7]. Specifically, between 15–60% of patients continue to progress despite adequate IOP control with medication, eventually about 9% become legally blind bilaterally [11, 12]. There is a critical and unmet medical need for neuroprotective therapies that protect RGCs and enhance their functionality to preserve and restore vision, as the majority of glaucoma patients already have suffered significant loss of RGCs at the time of diagnosis [5, 13, 14].

New therapeutic strategies that provide protection of RGCs will require the development of functional, reproducible, and easy-to-utilize animal models of glaucoma for preclinical studies. In our opinion, these models should meet the following criteria: (1) experimental insults mimic the glaucomatous pathology; (2) recapitulate the pathophysiological processes that appear during the course of human glaucoma; (3) shed light on the mechanisms underlying the death of RGCs; (4) provide sufficient degree of RGC death and axon degeneration in a reasonably short timeframe to enable efficient testing of neuroprotective therapeutics; last but not the least, (5) reproducibility. Inducing chronic IOP elevation is a prevalent approach for developing experimental animal models mimicking the pathology of POAG.

Currently available experimental rodent ocular hypertension (OHT) models however, as summarized in Table 1, have limitations due to one or multiple concerns: (1) low efficiency and high variability in inducing IOP elevation; (2) repeated procedures are often needed to maintain sustained IOP elevation which increase risks of complications, such as adverse effects from frequent anesthetization, cornea decompensation, injury to the iris or lens, intraocular hemorrhage, infection, etc.; (3) relatively long timeframe to cause significant RGC/axon loss (ie. most models need 6–10 weeks to result in 15–35% of RGC death); (4) the relationship between RGC/axon loss and ocular hypertension was poorly characterized.

**Table 1:** Summary of existing surgically-induced rodent ocular hypertension models.

Model (Procedure)	IOP profile	RGC death (mean)	Duration (weeks)	Pros	Cons	Reference
Cautery of episcleral vessels (aka Sharma model)	Unstable IOP elevation for more than 1–2 weeks	From ~15% (week 4) to ~40% (week 10) RGC loss	4–10	- Suitable for any genetic background. - Low risk of intraocular infection	- Surgery-associated risk of creating venous drainage of the choroid. - Hypoxia-mediated changes casts confounding effects to the retina unrelated to IOP elevation. - Neovascularization in the anterior chamber of episcleral vessels may compensate and enhance the drainage of aqueous humor.	[15–20]
Episcleral vein sclerosis (aka Morrison model)	IOP elevation is variable; subsequent injections are often needed to obtain sustained OHT in subset of animals	Highly variable	>4	- Suitable for any genetic background. - Low risk of choroidal damage - Low risk of intraocular infection	- Risk of ocular complications due to repeated procedures. - Low animal inclusion rate: only about 50% of the animals developed sustained IOP elevation after subsequent injections. - Low repeatibility and consistency from different batches of studies.	[21–25]
Laser occlusion of the episcleral vessels and/or trabecular meshwork	IOP elevation is variable; subsequent treatments are often needed to obtain sustained OHT	About 40–60% RGC loss	6	- Low risk of intraocular infection	- Risk of ocular complications due to repeated procedures. - Repeated laser treatments are often needed to maintain IOP elevation over the course of the study. - High individual variability in inducing IOP elevation.	[26, 27]
Circumlimbal suture	~50% of the sutured eyes maintained IOP elevation of 13% above baseline for 12 weeks	10% RGC loss	12	- Suitable for any genetic background - Low risk of intraocular infection	- Risk of ocular complications (ie >35% of eyes developed hyphema, 15% of eyes had suture breakage or conjunctival tear. - Only 50% of eyes showed chronic IOP elevation	[28]
Occlusion of aqueous outflow pathway by various materials: - polystyrene microbeads - polystyrene microbeads with the presence of viscous agent - magnetic microbeads	IOP elevation is variable and often transient with a duration of 1–2 weeks; subsequent injections are often needed to obtain sustained OHT	Varies from 5–25% of RGC loss from most reported studies	6–8	- Suitable for any genetic background	- High risk of intraocular complications due to repeated injections, such as cornea decompensation, injury to the iris or lens, intraocular hemorrhage. - Redistribution of the injected materials to the inferior quadrant of the anterior chamber shortly after the animals resuming activity, and clearance of the material from the ocular tissue overtime, resulting in insufficient blockage of the outflow pathway. - Small neurodegeneration window - Low animal inclusion rate: about 35–50% of the animals developed sustained IOP elevation after subsequent injections.	[29–33]

Taking all these aforementioned limitations into account, we have developed a novel approach to reliably induce chronic IOP elevation by a controllable manner. By utilizing a synthetic photopolymerizable biomatrix, hyaluronic acid glycidyl methacrylate (HAMA), and crosslinking the material at the iridocorneal angle using ultraviolet A (UVA) light to impede the aqueous outflow, sustained IOP elevation was induced after a single manipulation. This model for the most part recapitulates many of human glaucomatous characteristics including inner retinal thinning, RGC and axon loss, optic nerve degeneration and glial cell reactivity within one month after IOP elevation. This model may be considered as an *in vivo* tool for studying the pathophysiology of the disease onset and progression as well as for screening/validating neuroprotective therapies for treating glaucoma.

## Materials and methods

### Animals

We used C57BL/6J mice of either sex purchased from Jackson Laboratory (JAX, Bar Harbor, ME, US) that were typically 12–16 weeks old at the time of study. Mice used in a single experiment arrived at our research facility in a single shipment and were age-matched. Cages were randomly assigned to treatment and control groups with mice sharing a cage and receiving the same treatment (n = 4–5 mice/cage). Roughly equal number of males and females were used per study. All animal research described in this manuscript was approved by the Novartis Institutes for Biomedical Research IACUC Committee. Since bilateral ocular injections were performed in mice, adherence to the Association for Research in Vision and Ophthalmology Statement for the Use of Animals in Ophthalmic and Vision Research, necessitated that additional diligence towards their welfare was provided. Veterinary staff were consulted and initial pilot studies demonstrating minimal discomfort to mice with bilateral injections was ensured via monitoring of food/water intake as well as general behavior and health. Prior to the enucleation procedure, animals were euthanized by CO2 asphyxiation followed by confirmation of death via cervical dislocation.

### Preparation of injection set-up

The glass micropipette for ocular microinjection was made by pulling the glass capillary tube (World Precision Instruments, FL,USA, Catalog # 1B100-4) using a microelectrode puller (PC-10 puller, Narishige international, NY, USA). The tip of the micropipette was broken by forceps under surgical microscope to generate an opening of approximately 50 μm. The micropipette was coupled to a 22G needle connected to a 100μl Hamilton syringe using plastic PE tubing. The syringe and the tubing were filled with mineral oil (Sigma-Aldrich, St. Louis, MO, USA) to facilitate injection. Micropipettes were stored in sterilized petri dish until use.

### Synthesis of hyaluronic acid glycidyl methacrylate (HAMA)

A previously published protocol was adopted to synthesize HAMA [34]. In brief, 1 gram of HA (Lifecore, Cat No HA200k-5) was dissolved in 100ml of phosphate buffered saline (PBS) followed by slow addition of 100ml of N, N-dimethylformamide (DMF, Sigma, Cat No 227056). 50 fold molar excess of glycidyl methacrylate (GMA, Sigma, Cat No 0779342) was used to react with the pendant group of HA in the presence of 50 fold molar excess of trimethylamine (TEA, Sigma, Cat No 471283). The reaction was kept at room temperature for 5 days before purification by acetone precipitation and tangential flow filtration. Excess GMA, residual acetone and solvents will be removed by tangential flow filtration and lyophilization steps, the HAMA was characterized by nuclear magnetic resonance (NMR) to confirm the methacrylation ratio and purity. The final product was lyophilized and store at -80°C until further use. HAMA stock solution (4%) was made by reconstituting the dried HAMA powder in PBS and filtered through 0.22μm membrane to ensure sterility. Sterile aliquots were stored in 4 degree C fridge for up to 6 months until *in vivo* injection.

### Preparation of injection solution

4% HAMA was prepared in surgical grade sterile 1x phosphate buffered saline (PBS), plus 0.1% lithium phenyl-2,4,6- trimethylbenzoylphosphinate (LAP) as the photoinitiator. The stock solution was then filtered using vacuum driven filtration device (Millipore, SCGP00525). Polystyrene microbeads (15 μm in diameter, Cat. No. F8841, Life Technologies, Carlsbad, CA, USA) were centrifuged and re-suspended in surgical grade sterile 1x PBS at a concentration of 8x10^6^ microspheres/mL. Before injection, equal volumes of HAMA and microbeads solutions were mixed gently to result in a 2% HAMA-μBeads solution. Addition of microbeads into the HAMA solution allows for visualization during injection and for long term observation of the distribution of the HAMA gel after photopolymerization. HAMA-μBeads solution was protected from bright light in foil-wrapped Eppendorf tube and heated in water bath at 37°C degree before injection. Warming the solution at 37°C degree helped reduce the viscosity to facilitate injection.

### Intracameral injection and *in vivo* photopolymerization

**Fig 1:**
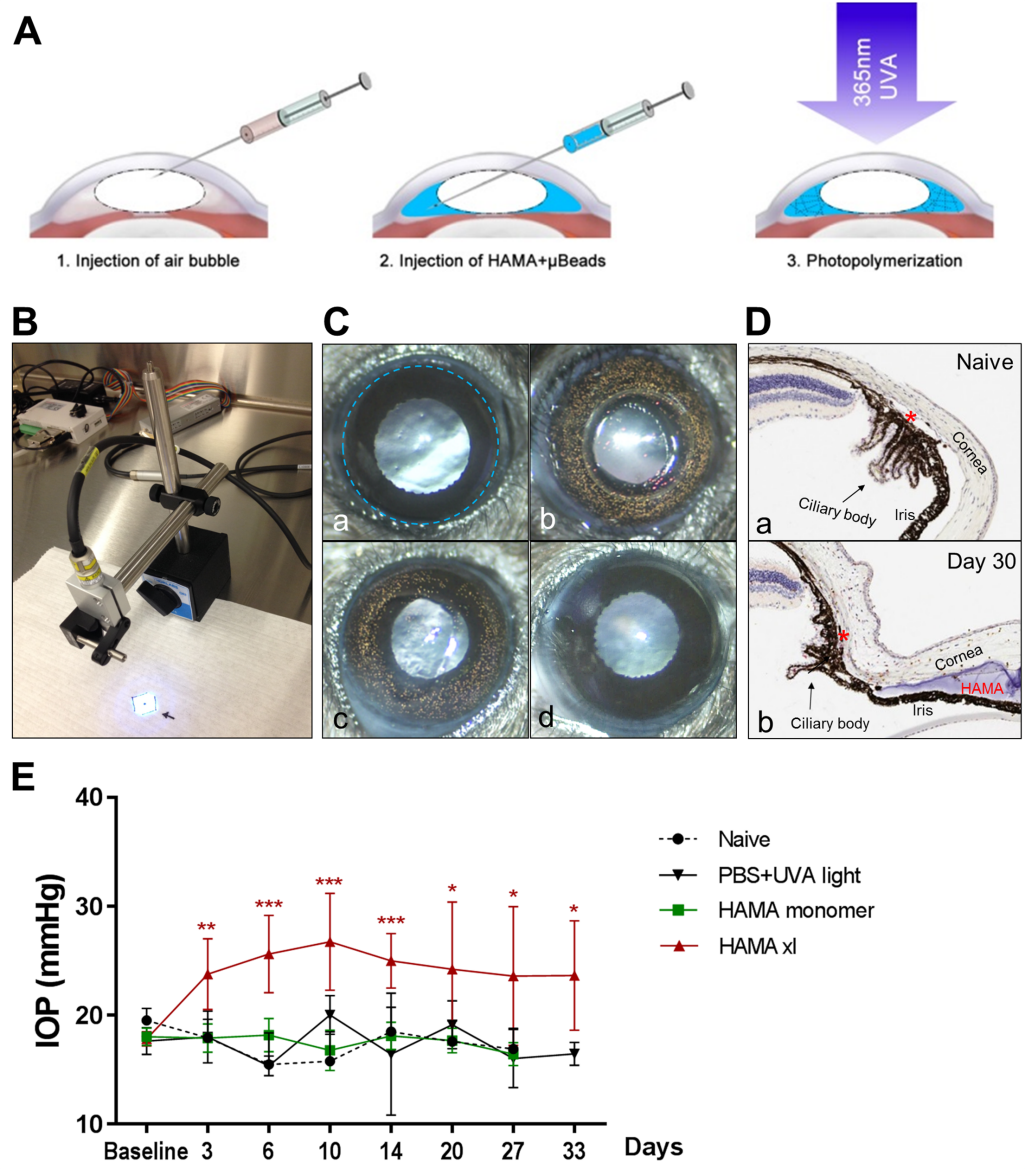
In vivo photopolymerization of HAMA-μBeads induced sustained IOP elevation for over one month. (A) Schematic indicating the microinjection of HAMA-μBeads into the anterior chamber and photopolymerization of HAMA at the iridocorneal angle to impede the aqueous outflow. 1. An air bubble (1μl) was first injected into the central anterior chamber via an opening made at the paracentral cornea. 2. 2% HAMA-μBeads solution (2μl, indicated as blue here) was injected into the interface between the air bubble and the aqueous humor. The air bubble guided the distribution of HAMA-μBeads to the iridocorneal angle and prevented efflux of the solution upon removal of the micropipette. 3. Immediately post-injection, HAMA was photopolymerized by defined UVA light at 365nm wavelength for 10 seconds, the μBreads were immobilized within the solidified HAMA gel for long-term tracking of the morphological change of the gel inside the anterior chamber. (B) UVA lamp that was programmed to generate UVA light at 365nm for 10 seconds per action. (C) Representative anterior chamber images before and after injection. a. pre-injection, the blue dotted circle marks the limbal region where the iris joins the cornea and sclera; b. shows the HAMA-μBeads ring formed along the iridocorneal angle immediately after photopolymerization; c. shows the distribution pattern of the HAMA-μBeads inside the anterior chamber 30 days post-injection, the HAMA-μBeads gel remained in place at the angle after 1 month post-operation; d. a HAMA monomer injected eye 30 days post-injection. (D) Hematoxylin and eosin stain of ocular vertical sections at the iridocorneal angle from a naive eye (a) and a HAMA-μBeads injected eye (b), the blue matter between the iris and the cornea in (b) is polymerized HAMA. Red asterisks indicate the position of the schlemm’s canal. (E) IOP profiles from the control and HAMA xl groups. Injection of 1XPBS followed by the UVA exposure (the PBS+UVA light group), or injection of HAMA monomer without the presence of μBeads and UVA crosslink did not cause IOP elevation. In vivo photopolymerization of HAMA-μBeads induced significant and sustained IOP elevation. n = 10–12 eyes/group for naive and PBS+UVA light groups, n = 44 eyes/group for HAMA monomer and HAMA xl groups. Group comparison was performed by one-way ANOVA followed by Tukey’s multiple comparisons (F = 26.11, P<0.0001); IOP elevation in the HAMA xl group at each time point was compared with the PBS+UVA light group by Student’s T-test, * P<0.05, ** P<0.01, *** P<0.001. Error bars indicate 95% confidence interval.

Mice were anesthetized with an intraperitoneal (IP) injection of a mixture of ketamine (100 mg/kg) and xylazine (10 mg/kg). Prior to injection, each eye was anesthetized with topical 0.5% proparacaine. Injection was performed under the Leica surgical microscope at low illuminant light (~6mW/cm^2^) in order to avoid prematurely polymerizing HAMA. The eyeball was immobilized by holding the temporal conjunctival tissue with a pair of microforceps. A small puncture in the paracentral cornea was made with a 32G bevel-tip needle, after taking out ~2.5 μl of aqueous humor using the glass micropipette, an air bubble (1 μl) was introduced into the central anterior chamber followed by injection of 2μl of 2% HAMA-μBeads solution at ~2μl/min speed using the micropipette (Fig 1**)**. The air bubble functions to guide the distribution of the HAMA-μBeads mixture along the iridocorneal angle forming a ring shape and seal the opening of the cornea to prevent efflux upon removal of the micropipette. The eye was either immediately exposed to a UVA light generator (LIGHTNINGCURE UV-LED module LC-L2, Hamamatsu Photonics, USA; holder purchased from Thorlabs, Inc., USA) for photopolymerization of HAMA at a distance of 10cm for 10 seconds with irradiance of 300mW/cm^2^ (Fig 1B) or not cross-linked (monomer control). Following the UVA exposure, the microbeads were immobilized into the polymerized HAMA gel to enable visualization and long term tracking of the HAMA gel in the anterior chamber. Same injection procedure was applied to the PBS and HAMA monomer groups, including the pre-administration of the air bubble. Eyes with PBS injection also received UVA light exposure. Neomycin ophthalmic antibiotic ointment was applied to all injected eyes.

### IOP measurement

IOPs were measured using a rebound tonometer (TonoLab; Tiolat, Oy, Finland) calibrated for use on mice eyes. Each IOP recording was the mean ± SEM of 10 successive readings. Animals were anesthetized with 3% isoflurane supplemented with oxygen at 2 liters per minute flow rate for 3 minutes, immediately followed by IOP measurement, a nose cone was attached to the animal for continuous isoflurane supply during IOP recording.

### Optical coherence tomography (OCT)

Ocular imaging was performed using a spectral domain OCT imaging system (Leica Microsystems, Bioptigen Envisu R2210). Images were acquired from C57Bl/6J mice (n = 7) prior to anterior chamber injection of HAMA/microbeads and 30 days following injection. Prior to imaging, pupils were dilated using 1–2 topical drops of 1.0% cyclopentolate hydrochloride (Alcon) followed by 1–2 drops of 2.5% phenylephrine hydrochloride (Akorn). Proparacaine 0.5% (Akorn) was also applied topically as a local anesthetic. Mice were anesthetized by intraperitoneal injection of a ketamine/xylazine (80.0 mg/kg /8.0 mg/kg) cocktail and subsequently maintained at sternal recumbency on a heating pad during imaging. Corneal hydration was maintained through application of 0.3% hypromellose lubricating drops (Novartis). OCT b-scans centered at the optic nerve were acquired in the inferior-superior and nasal-temporal directions from each eye. Ten 1.8 mm b-scans were acquired at each position and then subsequently aligned and averaged for analysis. Retinal thickness assessments were performed using custom code developed using MATLAB (Mathworks Release 2016a). Segmentation of retinal layers was performed manually by delineating the following areas: nerve fiber layer (NFL), posterior margin of the inner plexiform layer (IPL), anterior and posterior margins of the outer plexiform layer (OPL), and the RPE at Bruch’s membrane. The central 200 μm centered on the optic nerve head was excluded from the thickness measurements in order to avoid image alignment variability near the optic nerve. Retinal thickness measurements were made for each segment and averaged with measurements from the fellow eye. Total retinal thickness refers to the distance between the NFL and the RPE/Bruch’s membrane. Ganglion cell complex thickness refers to the distance between the NFL and the posterior margin of the IPL. Photoreceptor layer thickness refers to the distance between the posterior margin of the OPL and the RPE/Bruch’s membrane [= Outer nuclear layer (ONL) + inner segment (IS) + (outer segment) OS]. Data quantification and statistical analysis were performed using GraphPad Prism.

### Preparation of retinal flatmount and immunofluorescent staining

Mouse eyes were enucleated, and corneas were removed using iris scissors. Retinas were carefully dissected out from the eyecup, and cut at four quadrants towards the optic nerve head for flattening. Isolated retinas were fixed in 4% PFA for 30 minutes and washed in 1x PBS. Retinas were immunostained in primary and secondary antibody solutions free-floating for 48 hours and overnight respectively; flatmounts were washed with 1x PBS 5 times between and after antibody incubation, then flattened on slide with the ganglion cell layer facing up, briefly air-dried and mounted in anti-fade mounting medium (Cat. No. H-1500, Vector Laboratories, Burlingame, CA, US).

### Immunofluorescent staining of retinal section

Mouse eyes were harvested on Day 3 and Day 30 post injection, fixed in 4% paraformaldehyde (PFA) at 4°C overnight and processed for frozen sections. Ocular sagittal sections through the central retina at the optic nerve head position were obtained at 10 μm and 18 μm thickness for immunostaining. Indirect fluorescent immunostaining was performed for detecting antigens of interest using a pre-established protocol [35]. Briefly, sections were blocked in low protein IHC/ICC blocking buffer (Cat.No. 00-4953-54, Affymetrix eBiosciences, San Diego, CA, USA) at room temperature (RT) for 30min, followed by incubation with primary antibodies dissolved in 1x PBST (PBS+0.05% Tween-20) at 4°C overnight. Slides were washed in 1xPBS for three times then incubated in secondary antibodies at RT for 1 hour. The slides were washed and allowed to air dry before being coverslipped with anti-fade mounting medium with DAPI (Cat. No. H-1500, Vector Laboratories, Burlingame, CA, US). Information of antibodies used in this study was provided in Table 2.

**Table 2:** List of primary antibodies used for this study.

Antibody	Host	Working dilution	Catalog No.	Company and location
BRN3A	Mouse, monoclonal	1:100	MAB1585	Millipore, MA, USA
GFAP-cy3	Mouse, monoclonal	1:500	C9205	Sigma-Aldrich, MO, USA
IBA1	Rabbit, polyclonal	1:1000	019-19741	Wako laboratory Chemicals,VA, USA

Corresponding fluorescent secondary antibodies were all purchased from Life Technologies (Carlsbad, CA, USA).

### Optic nerve cross section and paraphenylenediamine stain

Mouse optic nerve samples were collected and fixed with half strength Karnovsky’s fixative (2% formaldehyde + 2.5% glutaraldehyde, in 0.1 M sodium cacodylate buffer, pH 7.4 (Electron Microscopy Sciences, Hatfield, Pennsylvania) for a minimum of 48 hours. After fixation, samples were rinsed with 0.1M sodium cacodylate buffer, post-fixed with 2% osmium tetroxide in 0.1M sodium cacodylate buffer, then dehydrated with graded ethyl alcohol solutions, transitioned with propylene oxide and resin infiltrated in tEPON-812 epoxy resin (Tousimis, Rockville, Maryland) utilizing an automated EMS Lynx 2 EM tissue processor (Electron Microscopy Sciences, Hatfield, Pennsylvania). Processed tissues were oriented in tEPON-812 epoxy resin and polymerized in silicone molds using an oven set for 60°C for 48 hours. Semi-thin cross-sections were cut at 1-micron with a Histo diamond knife (Diatome, Hatfield, Pennsylvania) on a Leica UC-7 ultramicrotome (Leica Microsystems, Buffalo Grove, IL) and collected on slides then dried on a slide warmer. The slides were stained with 2% aqueous paraphenylenediamine (MP Biomedicals LLC, Solon, Ohio) solution for 45 minutes at room temperature, rinsed in tap and deionized water solutions, air-dried, then mounting medium and a glass coverslip was applied over the sections for light microscopic analysis of myelinated axon analysis.

### Light and confocal microscopy

Retinal sections and flatmounts were examined and analyzed with a Zeiss LSM 700 confocal microscope (Zeiss, Thornwood, NY) equipped with argon (Ar) and helium/neon (He/Ne) lasers, using a Plan-APOCHROM 20×/0.8 M27 objective and an Axiocam HRm digital camera (Zeiss). Digital scans were acquired at the magnification zoom of 0.5 × to 2× and a resolution of 1024 × 1024 pixels. Confocal imaging was performed as stack scans at the interval of an optical thickness between 0.6 and 1.2 μm and 1.0 airy unit. For projections, typically 8–14 optical sections were acquired with an average total thickness of 6–16 μm and compressed for viewing. All confocal images shown are maximum projections of stacks. Digital confocal stacks were saved as Zeiss. LSM files and final publication quality images were exported in the .TIFF format at 300 dpi using Zeiss LSM 510 softwareZEN 2011. Images were adjusted for contrast and brightness, labeled, and formatted using Adobe Photoshop 7.0.1 (Adobe Systems, Inc., San Jose, CA) and saved at 300 dpi at their final magnification.

### Quantification of RGC and optic nerve axons

**Fig 2:**
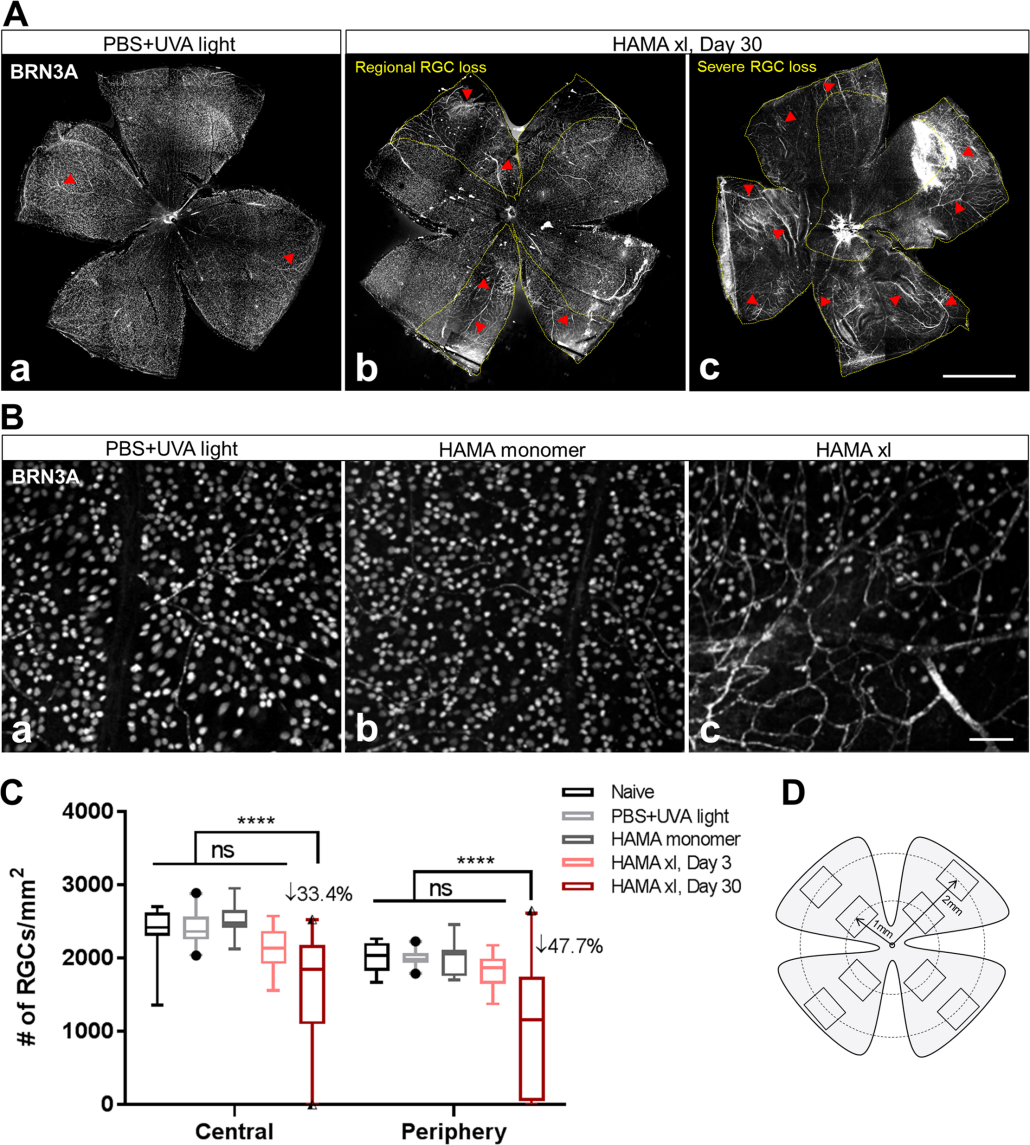
Ocular hypertension induced by HAMA xl led to significant loss of retinal ganglion cells (RGCs) one month post-operation. (A) Representative graphs of the Day 30 retinal flatmounts immunostained with BRN3A, a RGC nucleus marker. Loss of RGCs was observed from the hypertensive eyes treated with HAMA xl on Day 30. Note: the mouse monoclonal BRN3A antibody used in the present study cross-reacted with the blood vessels (red arrowheads) in the retina which became more visible in the degenerative retinas. Yellow dotted lines in b, c delineate the regions with more prominent RGC loss. Scale bar: 1 mm. (B) Representative micrographs of BRN3A immunolabeling of RGCs from normotensive (a. PBS+UVA light; b. HAMA monomer) and hypertensive (c. HAMA xl) retinas. Scale bar: 50 μm. (C) Quantification of RGC density based on BRN3A+ nuclei count from retinal flatmounts. Graph was shown as interleaved box & whiskers with 95% confidence interval. n = 11–12 eyes/group for naive, HAMA monomer (Day 30) and HAMA xl (Day 3) groups; n = 20 eyes/group for PBS+UVA light (Day 30) and HAMA xl (Day 30) groups, **** P<0.0001, ns: non-significant. Two-way ANOVA followed by Tukey’s multiple comparisons test. (D) Schematic indicating the sampling of eight 563μm x 422μm rectangle area in the retinal flatmount from four quadrants at two eccentricities (central vs periphery) from the optic nerve head (ONH) for RGC quantification (refer to S1 Fig for technical details).

**Fig 3:**
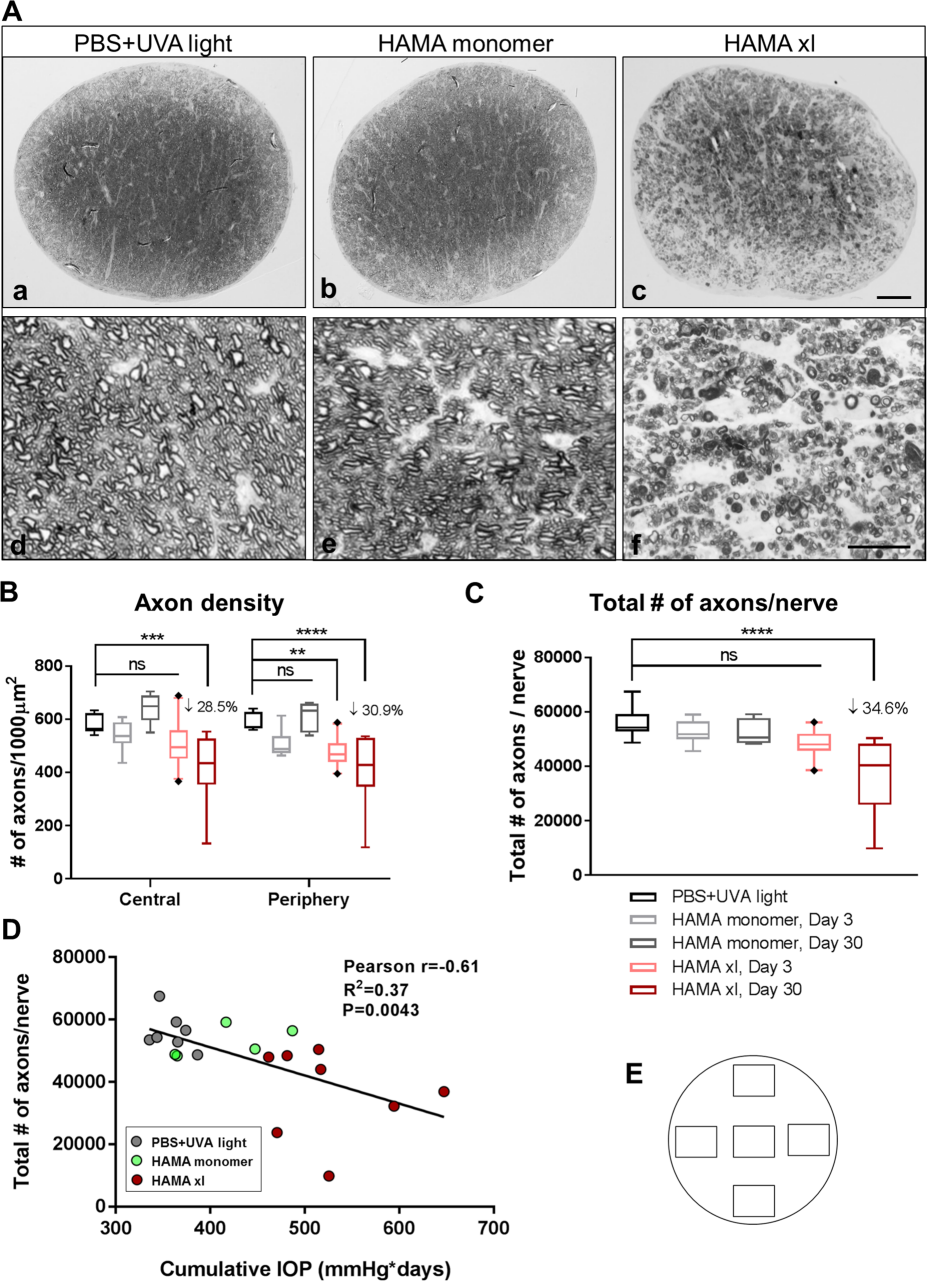
Ocular hypertension induced by HAMA xl led to significant degeneration of axons in the optic nerves one month post-injection. (A) Representative graphs of semi-thin optic nerve cross sections from Day 30 samples stained with paraphenylenediamine (PPD) at 200x (upper panels) and 1,000x (lower panels) magnifications. Scale bar in c: 50 μm; Scale bar in f: 10 μm. (B) Quantification of axon density in the central and periphery regions (refer to S2 Fig for technical details). (C) Quantification of total axon number per optic nerve. Graphs were shown as interleaved box & whiskers with 95% confidence interval. n = 5–8 eyes/group, ** P = 0.0031, *** P = 0.0002, **** P<0.0001. Statistical analyses were performed using Two-way (B) and One-way (C) ANOVA followed by Tukey’s multiple comparisons test (F = 6.808, P<0.0001). (D) Pearson’s correlation coefficient analysis of the association between total axon number and cumulative IOP on Day 30. P<0.01. The extent of axon loss appeared to be associated with cumulative IOP. (E) Schematic indicating five 110 μm x 82 μm rectangle area at the optic nerve cross section sampled for axon quantification (refer to S2 Fig for technical details).

To assess the degeneration of RGCs and their axons in hypertensive mouse eyes, we custom-developed automated RGC and axon quantification algorithms using CellProfiler and ImageJ, respectively (S1 and S2 Figs). RGCs were quantified on retinal flatmounts based on BRN3A immunostaining which specifically labeled the nuclei of RGCs. Eight 563μm x 422μm rectangle fields from four quadrants at two eccentricities from the optic nerve head (ONH) were sampled from each retina to quantify RGCs (as diagrammed in Fig 2D, refer to S1 Fig for more details). Axon degeneration was assessed by counting axons on optic nerve cross sections stained with paraphenylenediamine (PPD) to label myelin. Five rectangle areas (110μm x 82μm) from the optic nerve cross section (as diagrammed in Fig 3E, refer to S2 Fig for more details) were quantified for each sample.

### Quantification of astrocyte reactivity in the retina

The astrocyte reactivity was assessed based on the GFAP-immunoreactivity in the retinal flatmounts. One 563μm x 422μm rectangle field was sampled in the middle retina at 1.5mm away from the ONH at the superior, inferior, temporal and nasal retina, total of 4 fields were analyzed per flatmount, and the mean value was used for statistical analysis. The total area covered by GFAP-positive astrocytic soma and processes as well as the signal intensity was measured by HALO imaging software (Indica labs, Inc.).

Quantitative real-time Reverse transcription polymerase chain reaction (RT-PCR) was conducted to evaluate the mRNA levels of Gfap from the retinas. Briefly, total RNAs were purified using the Qiagen RNeasy Plus Mini Kit (Cat.No.74136, Qiagen, Germantown, MD), reverse transcribed into cDNAs using SuperScript III First-Strand Synthesis SuperMix (Cat.No. 18080400, Thermo Fisher Scientific, Waltham, MA). Quantitative PCR was conducted on the ViiA 7 Real-Time PCR System (Thermo Fisher Scientific, Waltham, MA) using the following primer pair for amplification of Gfap: forward primer: AGAAAGGTTGAATCGCTGGA; reverse primer: CGGCGATAGTCGTTAGCTTC.

### Quantification of microglia in the retina

Retinal vertical sections from the central retina were cut through the location of the optic nerve head and immunostained for ionized calcium binding adaptor molecule 1 (IBA1) to label microglia/macrophage. All microglia/macrophage with an identifiable soma were counted on the retinal sections; the total area of the retinal section was measured by ImageJ. The density of microglia/macrophage was calculated by the following formula: # of IBA1^+^ microglia/area of the retinal section.

### Statistics

Statistical analysis was performed using appropriate formulas embedded in GraphPad. Group values were expressed as mean ± SD or mean ± SEM as indicated in the text. Normally distributed data for IOP measurements from each group were compared by one-way ANOVA followed by Tukey’s multiple comparisons test. Pearson’s correlation coefficient was used for correlation analysis. The RGC and axon loss in control and experimental groups were compared using the Tukey's multiple comparisons test. Ocular hypertension induced retinal thinning measured by OCT was compared by paired Student’s t-test. GFAP-immunoreactivity was analyzed by Student’s t-test. Relative fold changes of GFAP mRNA in control and experimental groups were compared by multiple t-tests. Change of IBA1^+^ microglia density was analyzed by One-way ANOVA. A p-value <0.05 was considered statistically significant.

## Results

### Polymerization of HAMA at the iridocorneal angle induced sustained IOP elevation

Animals were randomly divided into four groups: (1) the OHT group received microinjection of 2% (w/v) HAMA mixed with microbeads (abbreviation: HAMA-μBeads) into the anterior chamber followed by *in vivo* UVA crosslink (referred to as the “HAMA xl” group hereafter); (2) one control group received PBS followed by UVA exposure (the “PBS+UVA” group); (3) another control group received injection of 2% HAMA without microbeads or UVA crosslink (the “HAMA monomer” group); and (4) a naive group with no treatment. Same injection procedure was applied for all three injection groups: removal of 2.5μl aqueous humor → administration of an air bubble (1μl) into the central anterior chamber → injection of 2μl solution (HAMA-μBeads, HAMA monomer, or PBS) → UVA crosslink for the HAMA xl and PBS groups (see “Intracameral injection and *in vivo* photopolymerization” in method for details). Administration of the air bubble served two important purposes, first it guided the distribution of the injected material to the iridocorneal angle forming a ring shape (Fig 1C); second it sealed the opening of the cornea to prevent efflux upon removal of the micropipette which was a common issue for intracameral injection via the cornea route. The presence of the air bubble did not cause IOP increase, as no IOP elevation was recorded in control or HAMA xl group immediately post-procedure (S1 Fig). The air bubble resolved gradually in 3–4 hours as observed during animal monitoring and the cornea opening had sealed by then.

Injection of the HAMA-μBeads solution following the pre-administration of an air bubble in the central anterior chamber guided the distribution of the material to the iridocorneal angle (Fig 1A and 1C). The HAMA was then immediately polymerized into a transparent gel form by exposing the cornea to a defined UVA light (365nm wavelength) at a distance of 10cm for 10 s (irradiance = 300mW/cm^2^), the microbeads were immobilized into the HAMA gel to enable long term visualization in the anterior chamber. The HAMA gel showed great stability and stayed in place at the angle after one month (Fig 1C-b,c and Fig 1D-b). We had observed very minimal degradation of the HAMA gel for up to 45 days post-injection. Being somewhat porous, crosslinked HAMA gel which was distributed along 360 degrees along the angle did not fully impede aqueous humor outflow but was sufficient to elevate IOP, which could be detected as early as 6 hours post-photopolymerization (S3 Fig). Sustained IOP elevation at average of 45 ± 20% (mean ± SD, same format hereafter unless stated otherwise) above baseline was noted for over 4 weeks (Fig 1E). The mean IOP in the HAMA xl group was 25.9 ± 3.6 mmHg compared to 17.4 ± 1.4 in the naïve group, 17.6 ± 0.7 in the HAMA monomer group, and 17.2 ± 1.6 in the PBS+UVA light group. IOP elevation in the HAMA xl group was statistically significant compared to the normotensive control groups (One-way ANOVA followed by Tukey’s multiple comparisons test, P<0.0001). In contrast, pre-administration of an air bubble followed by HAMA monomer or PBS+UVA light treatment did not cause IOP elevation (Fig 1E).

The HAMA material showed great biocompatibility, no complications were noted in any of the HAMA monomer injected eyes as well as the majority of the HAMA xl eyes (Fig 1C-c,d), in fact, this material has been used for tissue engineering, bioprinting and in vivo implant due to its similarity to natural hyaluronic acid, an essential component of the extracellular matrix [34, 36]. A small fraction of the HAMA xl eyes developed ocular complications (ranging 4–9% from 5 independent studies), such as persistent cornea haze or focal hemorrhage that did not resolve in 24 hours in the anterior chamber, in which case the animals were eliminated from the study. Using an *a priori* criterion of acceptable IOP elevation of ≥25% above baseline and absolute IOP not exceeding 60mmHg, plus no ocular complications, we experienced ~80% inclusion rate (ranging 77–85% from 5 studies) for the HAMA xl group. IOP greater than 60 mmHg were excluded in order to minimize the impact of high IOP in reduction of retinal blood flow that can result in retinal ischemia-induced loss of RGCs [37, 38].

### Chronic ocular hypertension caused significant RGC death and optic nerve degeneration

Changes to RGC numbers were assessed using immunofluorescent staining of retinal flatmounts with RGC marker, BRN3A (Fig 2A and 2B). Brn3a is a transcriptional factor specific to RGCs in the neuroretina that has been widely used to identify RGCs [39], our prior study had shown ~90% overlap of BRN3A-immunoreactivity with fluorogold injected into the superior colliculi to retrogradely label RGC soma (data not shown). HAMA xl induced OHT led to robust RGC death in 4 weeks, mean RGC density decreased by 33.4 ± 7.6% (mean ± SEM, same format hereafter) and 47.7 ± 10.0% on day 30 in the central and peripheral retina, respectively, compared to normotensive controls (P<0.0001 for both, two-way ANOVA followed by Tukey’s multiple comparisons test). HAMA monomer or PBS+UVA treatment, on the other hand, did not cause any significant RGC death compared to naive controls (Fig 2B and 2C). Furthermore, regional loss of RGCs was often observed in the hypertensive retinas as outlined by the dotted yellow lines in the representative retinas in Fig 2A-b,c. There was no significant RGC loss noted on day 3 in HAMA xl induced OHT eyes.

Consistently, 34.6 ± 9.0% of total axon loss was detected in the HAMA xl group on Day 30; whereas HAMA monomer or PBS+UVA light did not elicit axon degeneration compared to naive controls (One-way ANOVA followed by Tukey’s multiple comparisons test, P<0.0001, Fig 3A–3C). Apparent regional axon loss was observed in a subset of hypertensive nerves (S4 Fig), this regional difference was less notable in samples with overall severe degeneration (Fig 3A-f). In addition, no significant axon loss was detected on Day 3 in the HAMA xl group (Fig 3B and 3C). Correlation analysis revealed a positive relationship between total axon loss and cumulative IOP (Fig 3D, Pearson’s correlation analysis, P = 0.0043).

**Fig 4:**
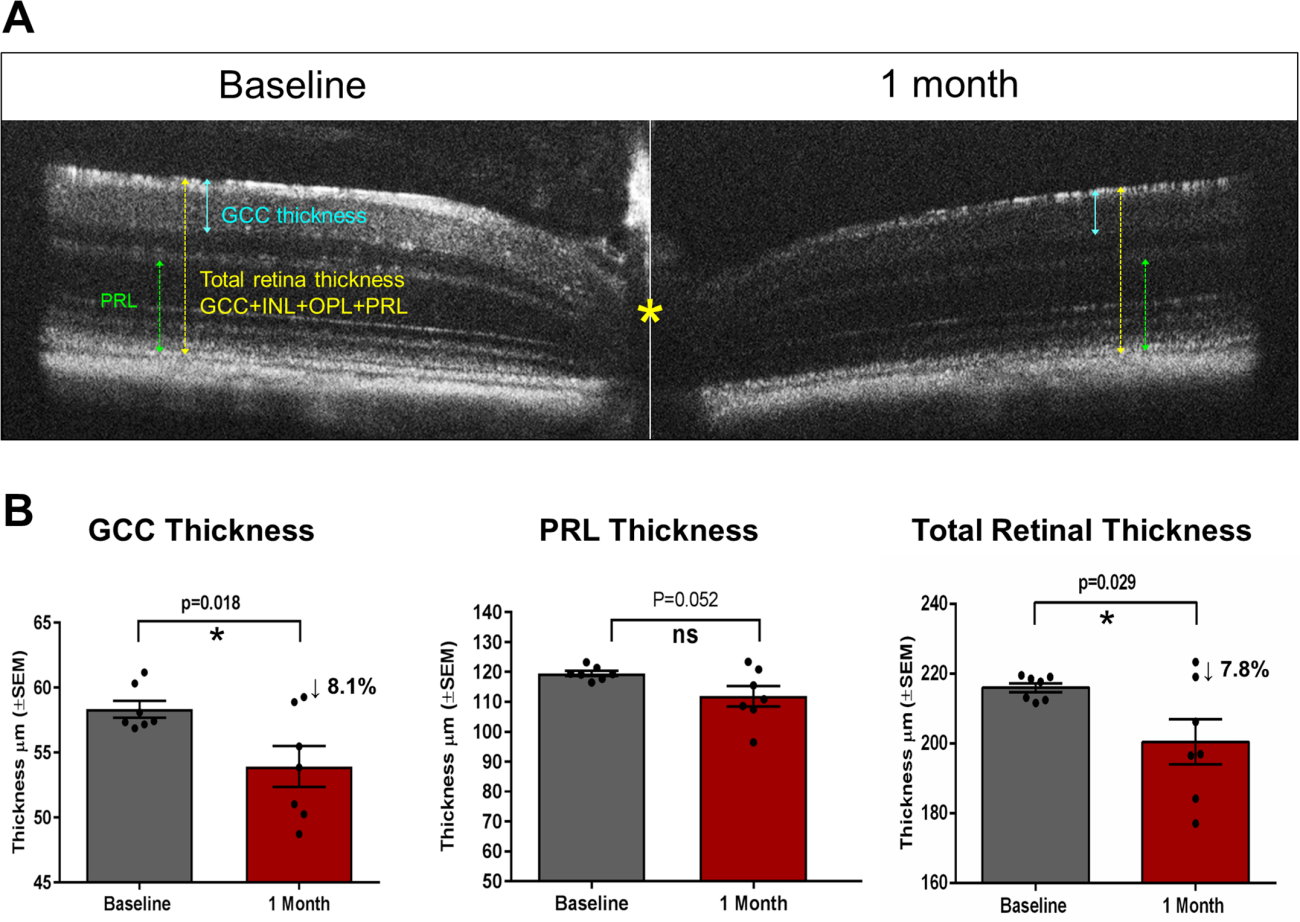
Ocular hypertension caused significant thinning of the inner retina. (A) Representative retina images obtained from optical coherence tomography (OCT) at baseline (left) and 1 month post-OHT induction by HAMA xl (right). Asterisk marks the position of the optic nerve head (ONH). (B) Analyses of the thicknesses of the ganglion cell complex (GCC), photoreceptor layer (PRL), and the total retina. GCC = NFL+ GCL+ IPL, as indicated by the blue solid line; PRL = ONL + IS + OS (from outer boundary of OPL to RPE), as indicated by the dotted green line; total neuroretina thickness was measured from NFL to RPE layer as indicated by the yellow dotted line. * P<0.05 by Student’s paired t-test.

**Fig 5:**
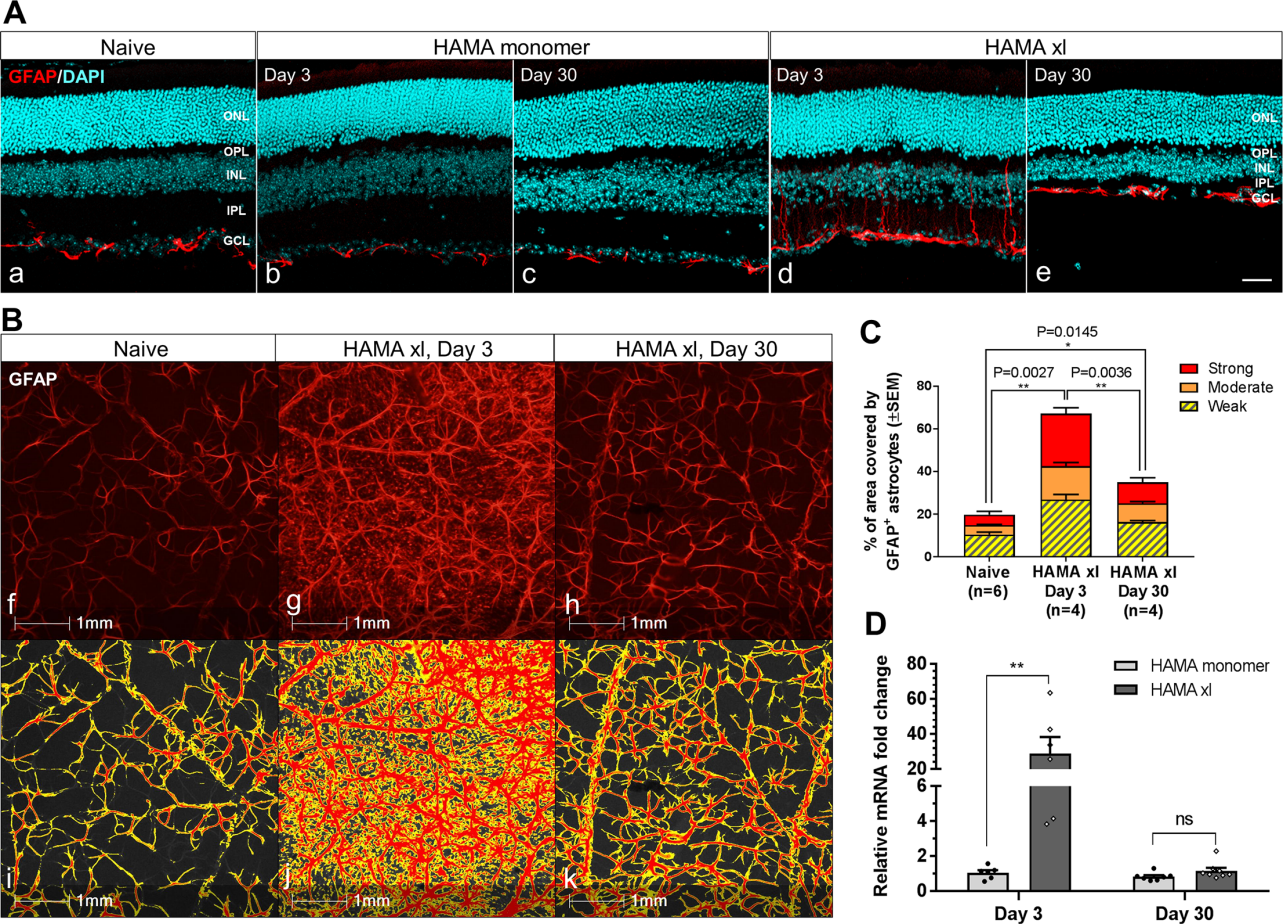
Astrocyte activation was observed in the hypertensive retinas with more prominent reactivity detected on Day 3 compared to Day 30. (A) Immunostaining of GFAP in retinal vertical sections. HAMA xl induced dramatic astrocyte activation with higher reactivity detected at early time point Day 3 (d) compared to Day 30 (e). In contrast, PBS+ UVA light (data not shown) or HAMA monomer (b,c) did not induce detectable astrocyte activation compared to naive controls. Images were collected from approximately 1.2–1.5mm from the optic nerve head in the retinal vertical sections. Scale bars: 50 μm. (B) Representative GFAP-immunoreactivity in retinal flatmounts. Top panel: GFAP immunostianing in retinal flatmounts from naive (f), hypertensive retina on day 3 (g) and day 30 (h); bottom panel: signals delineated by HALO software for corresponding images in f,g,h. No activation of astrocytes was noted in the retinal flatmounts from the PBS+UVA or HAMA monomer controls, data not shown. (C) Quantification of GFAP-immunoreactivity by HALO based on the total area covered by GFAP-immunofluorescence and the signal intensity. The intensity of GFAP-immunofluorescent signal was categorized as strong, moderate and weak by HALO. GFAP-immunoreactivity was significantly upregulated in the hypertensive retinas on Day 3 (P = 0.0027) and Day 30 (P = 0.0145) compared to naive controls. A significant attenuation of GFAP-immunoreactivity was also detected on Day 30 compared to Day 3 (P = 0.0036). Error bars: SEM. (D) GFAP mRNA was significantly upregulated in the hypertensive retinas on Day 3, which attenuated largely on Day 30. ** P = 0.008, multiple t-tests. Error bars: SEM.

**Fig 6:**
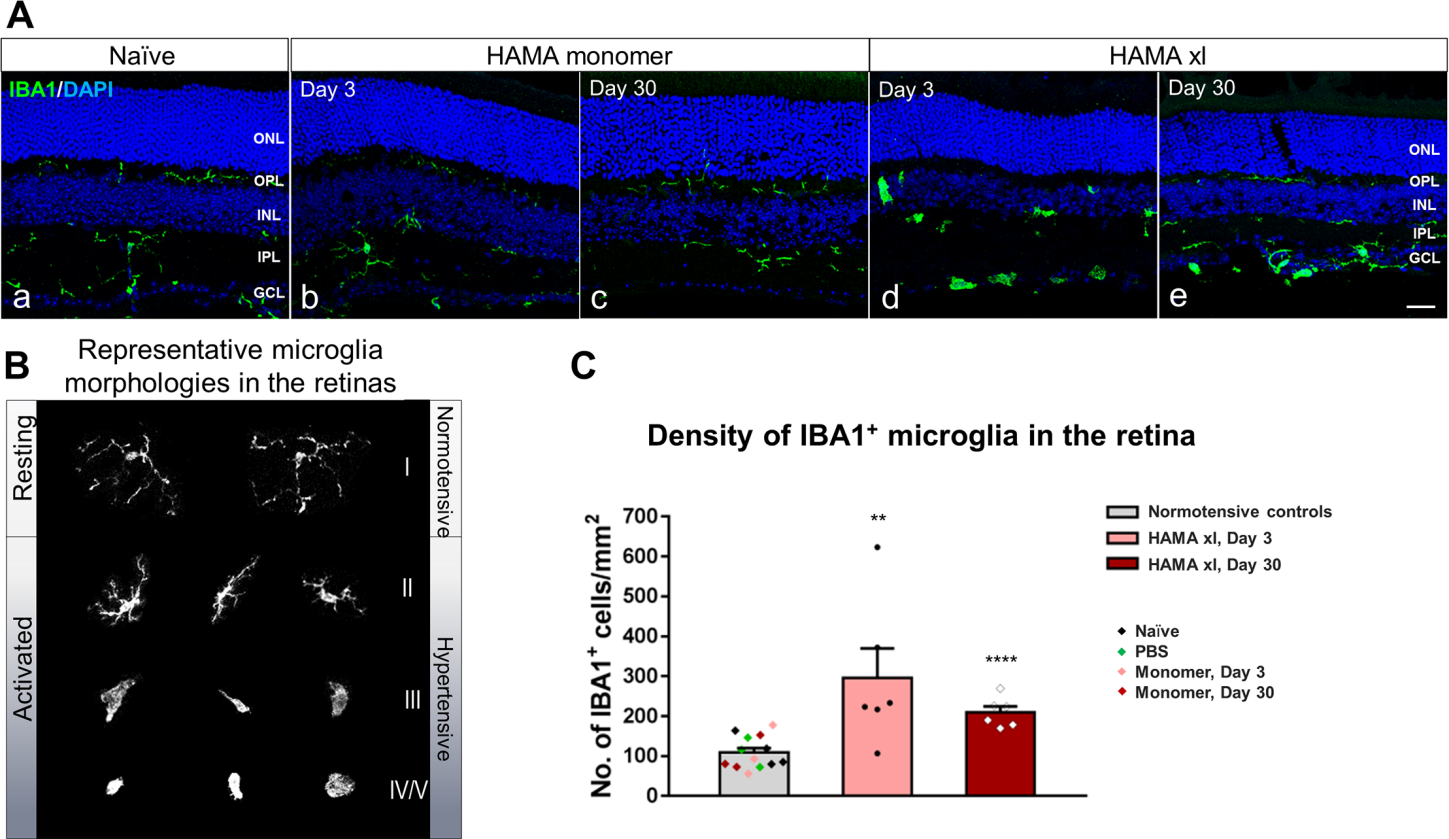
Ocular hypertension led to significant microgliosis in the hypertensive retinas. (A) Immunostaining of IBA1 to label microglial cells in the retinal vertical sections from normotensive (naive and HAMA monomer controls) and hypertensive (HAMA xl) retinas. Activation of microglia was observed in the hypertensive retinas on both Day 3 (d) and Day 30 (e). PBS+ UVA light (data not shown) or HAMA monomer (b,c) did not induce detectable microglia activation. Scale bar: 50 μm. (B) Representative microglia morphologies in the normotensive and hypertensive retinas. (C) Quantification of IBA1^+^ cells revealed significant increase of microglia cells in the hypertensive retinas on Day 3 and Day 30. One-way ANOVA. ** P = 0.0014, *** P<0.0001, error bars indicate SEM.

Furthermore, significant thinning of the inner retina, particularly the ganglion cell complex (GCC), was detected in the hypertensive eyes by non-invasive *in vivo* imaging Optical Coherence Tomography (OCT) (Fig 4). There was on average 8.1 ± 1.4% reduction on the thickness of the GCC–known as the sum of three innermost retinal layers: the nerve fiber layer (NFL), the ganglion cell layer (GCL), and the inner plexiform layer (IPL) (P = 0.018, Student’s paired t-test). The total retinal thickness (measured from the NFL to the retinal pigment epithelium layer) reduced 7.8 ± 5.5% (P = 0.029); the photoreceptor layer, defined as the outer nuclear layer (ONL) plus the inner segments (IS) and the outer segments (OS), was less affected (P = 0.052, Fig 4B). Loss of RGCs and thinning of the retina layers, particularly the inner retinal layers (NFL, GCL, IPL, and INL) in the hypertensive eyes, were also confirmed by qualitatively examining the retinal vertical sections; the thickness of the outer nuclear layer (ONL) was not significantly different from the normotensive controls (representative images in Fig 5A, Fig 6A).

### Ocular hypertension induced robust gliosis in the neuroretina preceding significant RGC death

Glial cell reactivity including the activation of astrocytes and microglia has been reported in human glaucoma [40–42] and various animal models of glaucoma [42–44]. However, the cellular changes of glial cells over the course of the disease onset and progression has remained poorly understood. To better understand the impact of OHT on glial cell reactivity and their changes in the process of RGC degeneration, we examined the astrocyte and microglia reactivity in the hypertensive retinas on Day 3 (before detectable RGC loss) and Day 30 (where significant RGC death has occurred) post the induction of IOP elevation.

Robust activation of astrocytes was observed in the hypertensive retinas when visualized with a well-characterized pan astrocyte marker, antibody against the glial fibrillary acidic protein (GFAP, Fig 5) [43, 45, 46]. In the healthy retina, quiescent astrocytes spread their processes laterally in the GCL (Fig 5A-a,b,c). Upon activation in response to OHT, the astrocyte cell body became enlarged with thickened processes. Prominent astrocyte reactivity was detected on Day 3, wherein activated astrocytes extended distinctive secondary processes directed vertically towards the outer retina (Fig 5A-d). Given the potential similarity to Müller cell processes, which are known to become upregulated following stress, we evaluated the possibility that these processes could be from activated Müller cells. However, there was little to no colocalization of GFAP with Cellular Retinaldehyde-Binding Protein (CRALBP), a well-established Müller cell marker (S5 Fig, thus the vertically extending GFAP-positive processes were mostly from activated astrocytes. In comparison, the astrocyte reactivity appeared attenuated and confined to the ganglion cell layer on Day 30 (Fig 5A-e), although still remained activated compared to that in the normotensive control retinas. Higher magnitude of astrocyte reactivity on Day 3 was also apparent in the hypertensive retinal flatmounts with greater GFAP-immunoreactivity and more area covered by GFAP-positive processes compared to those on Day 30 (Fig 5B and 5C). No activation of astrocytes was noted in the retinal flatmounts from the PBS+UVA or HAMA monomer controls (data not shown). Consistently, significant upregulation of *Gfap* mRNA was detected in the hypertensive retinas on Day 3 by an average of 28.9 ± 9.4 fold compared to the that of the HAMA monomer retinas (P = 0.008, multiple t-tests) by quantitative real-time reverse transcription-polymerase chain reaction (qRT-PCR). In contrast, no significant difference in *Gfap* mRNA levels on day 30 was detected (Fig 5D), however the GFAP-positive astrocytic processes remained elevated as seen in retinal vertical sections and flatmounts. HAMA monomer or PBS+UVA light did not elicit significant *Gfap* mRNA changes compared to naive controls (data not shown).

Both resting and activated microglia can be identified by immunostaining of the ionized calcium binding adaptor molecule 1 (IBA1, Fig 6). In the normotensive retina, resting microglia were primarily localized in the inner and outer plexiform layers which bear multiple long ramified fine processes radiating from a small somata (Fig 6A-a,b,c). In the hypertensive retinas, activated microglia became less ramified or completely lost their processes, some cells transformed into the amoeboid or round/rod-shaped morphology, and their cell bodies were prominently enlarged compared to those at the resting state (Fig 6A-d,e; 6B). Quantification of the IBA1-positive cells revealed a significant increase in microglia density in the hypertensive retinas—about 3-fold increase on Day 3 (2.7 ± 0.7, P = 0.0014) and 2-fold on Day 30 (1.9 ± 0.1, P<0.0001) compared to normotensive controls (One-way ANOVA, Fig 6C). Interestingly, we observed a trend towards higher number of amoeboid- (type III in Fig 6C), round/rod-shaped or “gitter” microglia (type IV/V) presented in the GCL and the INL in the hypertensive retinas on Day 3 compared to Day 30, whereas reactive microglia with enlarged soma and retracted and thickened “bushy” branches were more frequently seen (Fig 6A-d,e). Note that although IBA1 has been widely used for labeling retinal microglia, it does not distinguish resident retinal microglia from infiltrating macrophages or monocytes. Therefore, the quantification did not exclude infiltrating macrophages/monocytes if there were any.

## Discussion

A range of methods have been developed to experimentally induce OHT in various animal species to mimic human condition of OHT, including monkeys, dogs, cats, rats, mice, birds and zebra fish (review article by Bouhenni, et al. 2012)[22]. The predominance of using rodent models is based on their similarities to humans with respect to the anatomical and developmental features of the ocular anterior segment, aqueous humor circulation, ease of genetic manipulation, and optic nerve changes caused by IOP elevation [26]. Valuable information on the molecular and cellular mechanisms underlying glaucomatous RGC death have been obtained from animal models, however, various challenges also exist with the currently available *in vivo* models. Few common concerns include high variability, low success rate in inducing sustained IOP elevation and repeated manipulations needed to maintain chronic OHT, which in turn result in higher incidence of complications (Table 1). For instance, our experience with the commonly used polystyrene microbeads model [29, 30] showed that the microbeads redistributed to the inferior quadrant of the anterior chamber due to gravity shortly after the animal recovered from anesthesia, resulting in insufficient occlusion of the outflow. IOP elevation that lasted >1 week was only obtained in less than 50% of the animals (data not shown). Furthermore, the aggregation of microbeads in the inferior (ventral) region often caused focal hemorrhage and corneal synechiae to >50% of the injected eyes (unpublished in-house observation) potentially confounding the mechanisms in neurodegeneration. Modified approaches have been reported by different groups, such as mixing the microbeads with viscoelastic solution [31] or using magnetic microbeads and a handheld magnet [33] to prevent microbeads reflux after injection and improve the bead distribution in the anterior chamber, however, the effects were only short term and subsequent re-injection was needed for a subset of animals. A circumlimbal suture approach has recently been reported by Zhao and colleagues (2017) where circumlimbal suture was applied at 5–6 subconjunctival anchor points behind the limbus to induce chronic IOP elevation in mice [28]. Whilst this approach had the advantage of not introducing foreign materials into the eye, the success rate was still around 50% and over half of the eyes showed complications including hyphema and suture breakage, slippage or conjunctival tear.

In order to improve the success rate and reliably inducing chronic OHT, we have developed a novel approach to induce sustained IOP elevation in the mouse eye at a more controllable manner using the synthetic biomatrix HAMA combined with an air bubble injection technique. By *in vivo* photopolymerization of the HAMA at the iridocorneal angle, sustained IOP elevation was induced after a single procedure. HAMA, a modified biomatrix using the hyaluronic acid as backbone, has shown great biocompatibility and stability inside the eye following an intracameral injection. This approach has shown high repeatibility in inducing sustained IOP elevation (refer to S6 Fig for representative IOP curves from three repeated studies).

In the present model, we observed ~35% of RGC and axon loss after 4 weeks of sustained IOP elevation at 45% above baseline IOP. The ability of this model to demonstrate RGC loss at ~25 mmHg is comparable to that shown by Chen et al. (2011) and Gross et al. (2003) [29, 47]. However unlike the present findings, McDowell et al (2012) did not demonstrate a reduction in RGCs following Ad5.MYOC.Y437H-mediated OHT for up to 8 weeks [48]. The reasons for this discrepancy could be attributable to several factors including possibly the consistency in maintenance of sustained IOP elevation over the duration of the experiment.

The degree of RGC death and axon degeneration in the present model is comparable to other experimental glaucoma rodent models. For instance, most ocular occlusion models reported 10–25% of RGC death in 2–6 months [17, 18, 20, 29, 30, 33, 49, 50]; comparable RGC loss (30–40%) in 1.5–6 months was also reported with the hypertonic saline model [51, 52] and the episcleral vein cautery model [18, 53, 54], with reported peak IOP elevation ranging from 1.6–2.5 fold above baseline in these OHT models. In addition, we observed that the RGC loss often displayed regional or sectorial pattern in the hypertensive retinal flatmounts (Fig 2A). In agreement with our observations, clustered or sectorial RGC loss has been reported in other rat ocular hypertension models [55]. The mechanisms underlying the regional RGC loss remain to be elucidated. One plausible speculation is that the degenerating RGCs express damage-associated molecular patterns, DAMPs [56, 57], leading to locally amplified inflammation and glial cell reactivity, which in turn play a role in mediating regional RGC loss. Additional characterizations of the molecular pathways resulting the RGC loss in this model are being performed.

Like all animal models, limitations exist in this model as well. Firstly, although mice share many similarities with human on the anatomical, developmental and genetic features of the ocular structure, it is lacking a true collagen-rich laminar cribrosa; instead, a glial lamina is present at the optic nerve head region [58, 59]. We did not detect prominent cupping of the optic nerve head, a hallmark of glaucoma. Secondly, the present model is developed with the intention for studying glaucomatous neurodegeneration and for testing potential neuroprotective therapies; like other anterior chamber occlusion models, this model may not be suitable for studying IOP lowering agents with the mechanisms of action targeting on enhancing conventional aqueous outflow. Moreover, HAMA gel did not cross link with the iris and did not prevent pupillary constriction/dilation responses, however, we have noticed a slower response of pupillary dilation to dilation drops (1% cyclopentolate, followed by 10% phenylephrine). Some HAMA xl treated eyes displayed less dilation compared to the healthy control eyes. While this reduced dilation was not an issue for OCT imaging, however it may require appropriate normalization approach when evaluating visual function changes by electroretinogram (ERG) or optokinetic motor response. Furthermore, glaucoma is a multifactorial heterogeneous neurodegenerative disease; this model may not recapitulate all pathophysiological features that happen in human glaucoma, especially for normal tension glaucoma.

Accumulating evidence supports an active role of glial cells in mediating the inflammation and pathogenic process of the glaucomatous neurodegeneration. In glaucomatous neuropathy, resident glia in the retina and the optic nerve head (ONH) alter their gene expression profile during “activation” state, likely exerting neuroprotective or neurodestructive influences at different phases of disease process [45, 60–65]. Altered crosstalk between RGCs and glial cells has been proposed as early factors leading to the pathology of glaucoma [46, 66]. Interestingly, in the HAMA xl model we noticed dramatic and distinctive temporal reactivity changes of activated astrocytes and microglia in the hypertensive retinas. At the early time point on Day 3, the activated retinal astrocytes underwent profound morphological remodeling and extended very distinctive secondary processes. Many of these processes projected vertically towards the outer retina. The magnitude of reactivity, however, was attenuated during the “chronic” reactive phase with overall lesser secondary processes and fewer to no vertically extending processes seen on Day 30. Although the lateral processes in the GCL still remained thickened, indicative of a different activation state that were more confined to the GCL, implying a role of chronically activated astrocytes in limiting the degenerative events locally. Concurrently, there was a dramatic upregulation of GFAP-immunoreactivity and *Gfap* mRNA in the hypertensive retina on Day 3 which declined on Day 30. There were few studies on the early responses of astrocytes in the hypertensive retina; on the other hand, acute and reversible astrocyte reactivity in the optic nerve head has been extensively studied primarily in the anterior chamber ischemia models with short-term IOP elevation by anterior chamber cannulation [67, 68]. For instance, Morrison’s group reported that the ONH astrocytes rearrange their extensions immediately after elevating the IOP to 60mmHg for 8 hours and re-orientated back to baseline orientation 1 day post IOP normalization [68]. Whereas, Sun et al. (2013) reported that IOP elevation to 30mmHg for 1 hour induced significant remodeling of astrocytes at the ONH which peaked on Day 3 and returned to resting state at 6 weeks [67]. The temporal reactivity patterns of the retinal astrocytes observed in the present study added additional knowledge on the behavior of reactive astrocytes in response to chronic OHT in the neuroretina. Our data suggested that retinal astrocyte activation is an early event to IOP elevation preceding significant RGC death. The distinctive temporal reactive patterns of astrocytes at the acute and chronic phases of ocular hypertension imply that the reactive retinal astrocytes may play different roles in mediating the onset and progression of glaucomatous RGC degeneration. In fact, recent studies from Barres’ group (2017) demonstrated that different initiating CNS injuries could elicit at least two types of ‘‘reactive” astrocytes with strikingly different properties, one type (namely A2 astrocyte) being helpful and the other pathologic (A1 astrocyte) to neurons, these two types of reactive astrocytes presented distinctive transcriptome profiles [69, 70]. Additionally, astrocyte proliferation is also noted in various glaucoma rodent models [46, 71] but not in DBA2J mouse model of glaucoma [43, 72]. Whether astrocyte proliferation occurs in the present model remains to be determined. Reactive astrocytes in the hypertensive retina may be neuroprotective at the early disease onset phase and could become neurotoxic resulting in progressive RGC death, however this remains to be further elucidated in this model.

Microglia are CNS-resident innate immune cells, endowed with sensor and effector functions as well as with phagocytic capacity during physiological and pathological conditions [73–75]. In human glaucoma, there is abnormal microglia reactivity and redistribution within the ONH, where optic nerve pathology is first detectable [41, 75, 76]. Likewise, reactive microgliosis is detectable in retinae and severely damaged nerves from animal models of chronic glaucoma [72, 77] and of induced ocular hypertension [18, 78]. However, the mechanisms controlling microglial recruitment and activation in human or animal models of glaucoma have not been established, moreover, it is unclear whether and how reactive microglia undergo functional changes over the course of disease progression. In the present study, we observed overall strong microglial activation at the disease onset stage preceding significant RGC death. Microglia at late activation states with the amoeboid- (type III), round/rod-shaped or “gitter” morphologies (type IV/V) were most frequently detected in the GCL and the INL in the hypertensive retinas on Day 3, compared to Day 30 wherein reactive microglia with enlarged soma and retracted and thickened “bushy” branches (type II) were more frequently detected in the GCL (Fig 6B). The higher degree of microgliosis observed at the early stage of the degenerating retina coincided with the temporal relativity changes of activated retinal astrocytes. This is not surprising since reactive astrocytes can be induced by activated microglia [69, 70]. In agreement with our observation, a study by Banati (2003) reported that in acute lesions in the CNS the peak of microglial activation had occurred 2–3 days post insult, but if the pathological stimulus persisted then microglial activation continued [79]. Furthermore, Bosco et al. (2011) and others reported that a peak of microglia clustering and IBA1 expression in the central retina and optic nerve head (ONH) was detected in the chronic inherited DBA2J mouse glaucoma model at 3 months of age, preceding detectable RGC degeneration [80–82]; by 12 months, microglial cells were drastically reduced in their numbers and levels of IBA1 expression were decreased in the retina and ONH.

It has been generally agreed that activated microglia are able to migrate to the site of injury and phagocytose damaged cells and display the resulting immunomolecules. Phagocytic microglia secrete pro-inflammatory factors (ie. IFN-γ, IL-1α, IL-1β, TNF-α, and nitric oxide) to promote more microgliosis. Activated phagocytic microglia also interact with astrocytes and RGCs as quickly as possible with minimal damage to the healthy cells [83, 84]. One concern however is that IBA1 is not a selective marker for microglia and can also label infiltrating macrophages. So it is possible that the observations made herein could include infiltrating macrophages and resident microglial cells.

Collectively, we observed greater glial reactivity at the disease induction stage preceding significant RGC death, suggesting glial cell activation is an early event in glaucomatous neurodegeneration. The time course of the glial cell reactivity observed in this model is intriguing. How do these different reactivity patterns reflect to glial cell functions and gene expression in the disease onset and progression process? Are acute glial cell responses neuroprotective [85]; and become detrimental with the persistence of the pathological insults? If so, what are the key factors that drive such functional switches? Can we develop therapeutics that modulates the functions of glial cells for neuroprotection? The establishment of this new glaucoma model may facilitate answering these questions, and may be used to support the development of novel neuroprotective therapies for treating glaucoma as well as other CNS neurodegenerative disease.

## Supporting information

S1 Fig Diagram indicating the quantification of RGCs on retinal flatmounts based on brn3a immunostaining. Left: schematic indicating the sampling of eight 563μm x 422μm rectangle area in the retinal flatmount from four quadrants at two eccentricities from the optic nerve head (ONH) for RGC quantification; right: example of counting Brn3a^+^ RGC nuclei by custom-developed algorithm in CellProfiler, the blood vessels that were unspecifically labeled by Brn3a (purple) were filtered out by the algorithm, only RGC nuclei (green) were counted.
(TIF)

S2 Fig Diagram indicating the quantification of axons in the optic nerve cross sections by PPD stain. A. Five 110 μm x 82 μm rectangle area at the optic nerve cross section were sampled for axon count as shown in the upper left diagram. The number of axons was counted by custom-developed algorithm using ImageJ based on PPD-staining of myelin.
(TIF)

S3 Fig IOPs before and after anterior chamber injection. IOPs were measured immediately before (~1min) and after injection + crosslink of HAMA or PBS (~1min), and again at 6 hours post-injection. PBS+UVA light: n = 14; HAMA xl: n = 29. **** P<0.0001, Student’s T-test, error bars indicate SEM.
(TIF)

S4 Fig Example of regional axon loss in a hypertensive optic nerve. A. Optic nerve cross section stained with paraphenylenediamine (PPD) at 200x magnification. Red arrows point to a region with severe axon loss. Scale bar: 50 μm. B. Micrograph from A taken at 1,000X magnification. Scale bar: 10 μm.
(TIF)

S5 Fig **Co-immunofluorescnet staining of a hypertensive retina (Day 3) with CRALBP (a) and GFAP (b).** GFAP (green) signal did not co-localize with CRALBP (red), a Müller cell marker. Blue color in (C) indicated DAPI stain.
(TIF)

S6 Fig Comparison of IOP curves from three independent studies. Shown here are IOP elevation curves from one additional study using 2% HAMA + microbeads, and two independent studies using 2% HAMA alone. Y axis indicates fold of IOP elevation relative to corresponding mean IOP of (PBS+UVA light) controls. n = 10–13 for control groups/study. The presence of microbeads did not impact IOP elevation. P = 0.66, Two-way ANOVA.
(TIF)
