# Supplementary figures and images for: A murine glaucoma model induced by rapid in vivo photopolymerization of hyaluronic acid glycidyl methacrylate
Source: PLoS One. 2018 Jun 27;13(6):e0196529. doi: 10.1371/journal.pone.0196529 (PMC6021085; doi:10.1371/journal.pone.0196529)

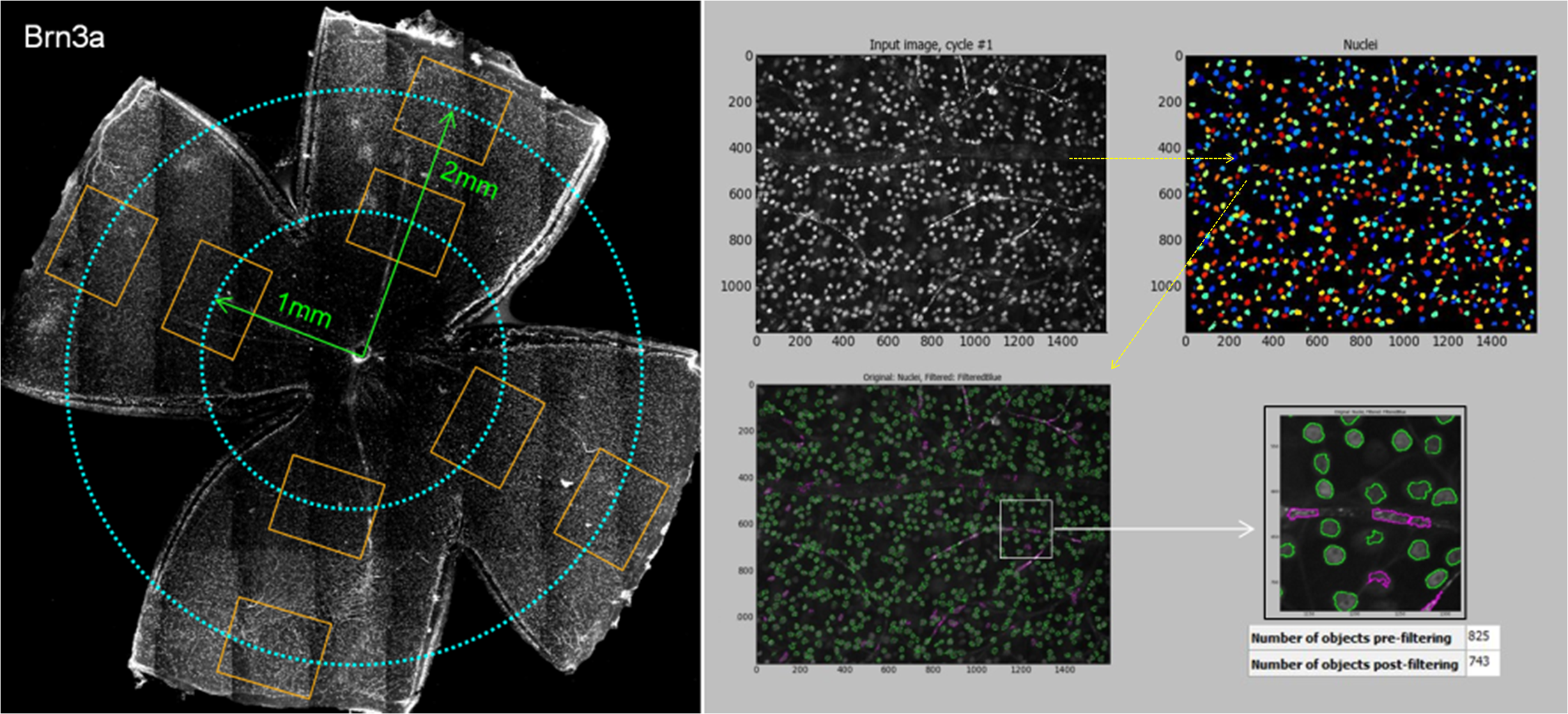

Supplement: S1 Fig — Left: schematic indicating the sampling of eight 563μm x 422μm rectangle area in the retinal flatmount from four quadrants at two eccentricities from the optic nerve head (ONH) for RGC quantification; right: example of counting Brn3a+ RGC nuclei by custom-developed algorithm in CellProfiler, the blood vessels that were unspecifically labeled by Brn3a (purple) were filtered out by the algorithm, only RGC nuclei (green) were counted. (TIF) [file pone.0196529.s001.tif]

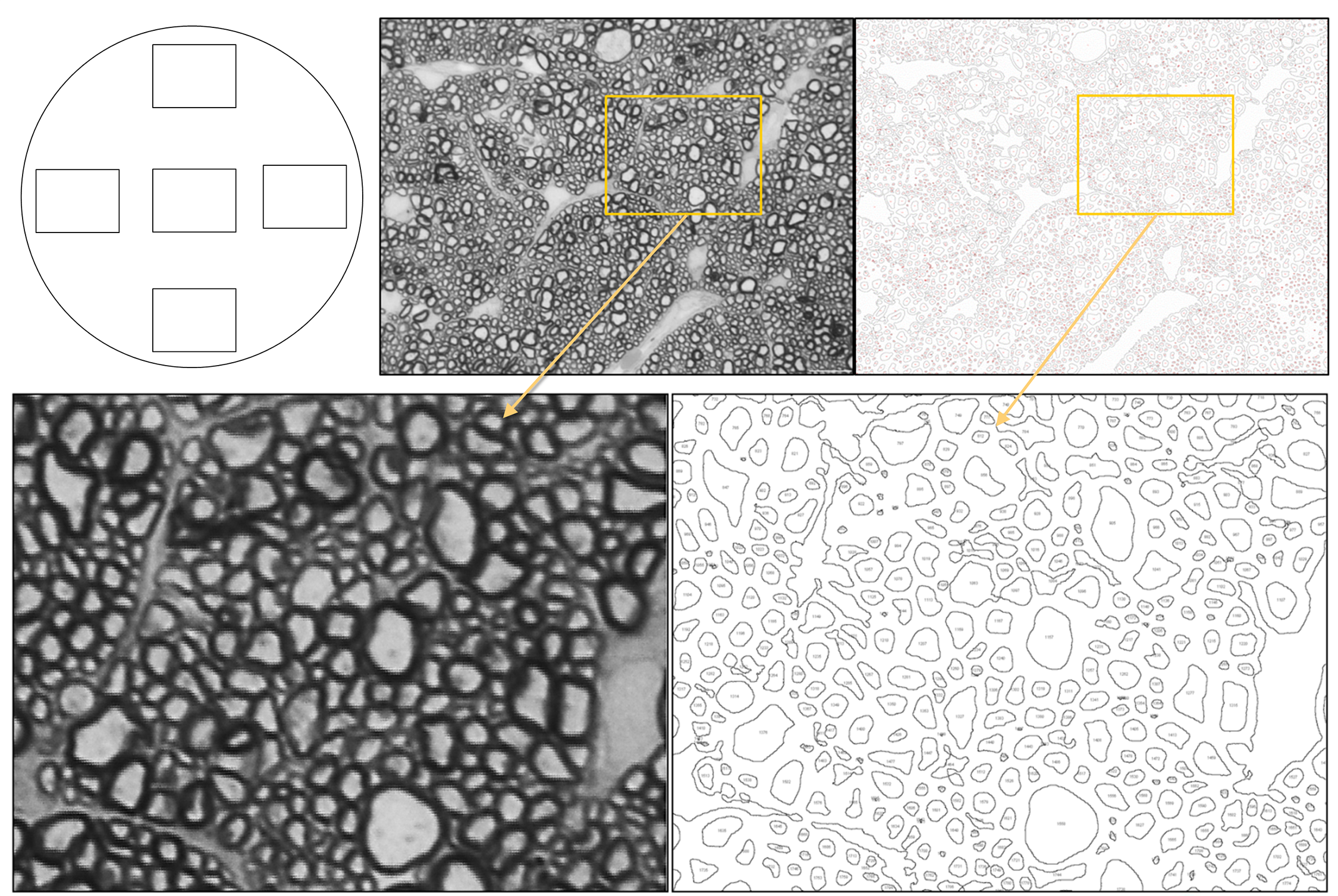

Supplement: S2 Fig — A. Five 110 μm x 82 μm rectangle area at the optic nerve cross section were sampled for axon count as shown in the upper left diagram. The number of axons was counted by custom-developed algorithm using ImageJ based on PPD-staining of myelin. (TIF) [file pone.0196529.s002.tif]

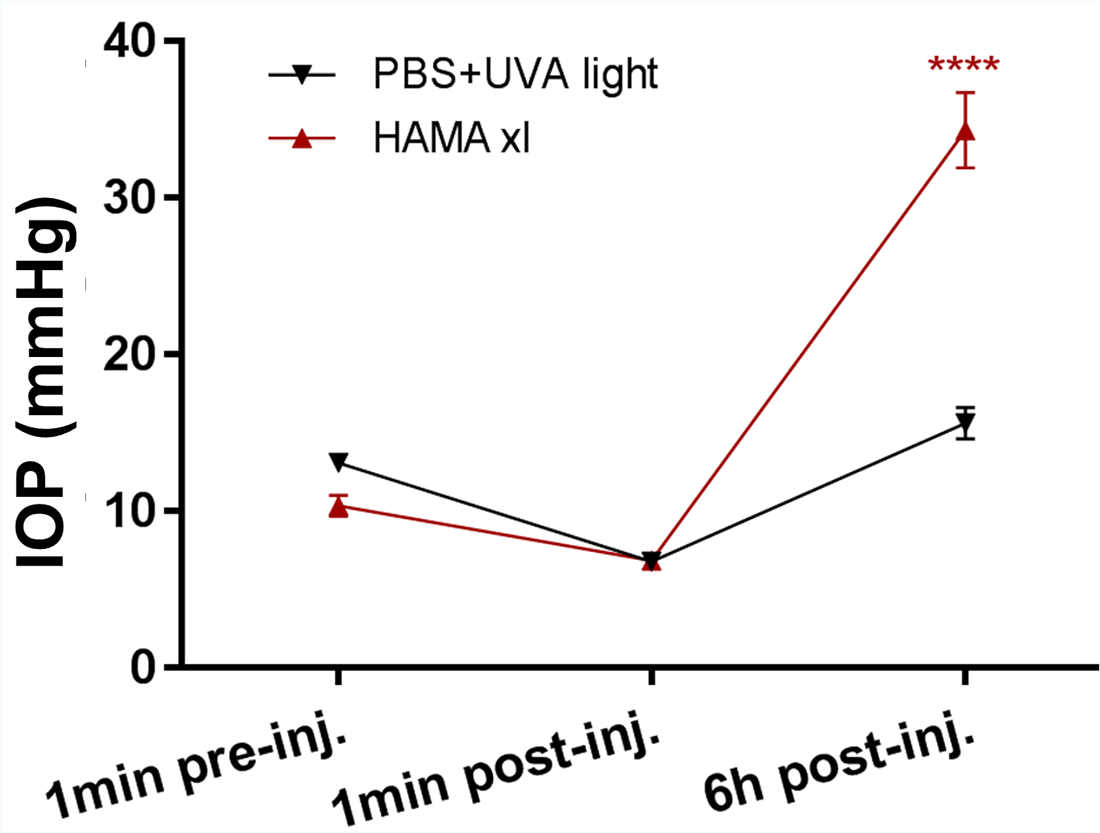

Supplement: S3 Fig — IOPs were measured immediately before (~1min) and after injection + crosslink of HAMA or PBS (~1min), and again at 6 hours post-injection. PBS+UVA light: n = 14; HAMA xl: n = 29. **** P<0.0001, Student’s T-test, error bars indicate SEM. (TIF) [file pone.0196529.s003.tif]

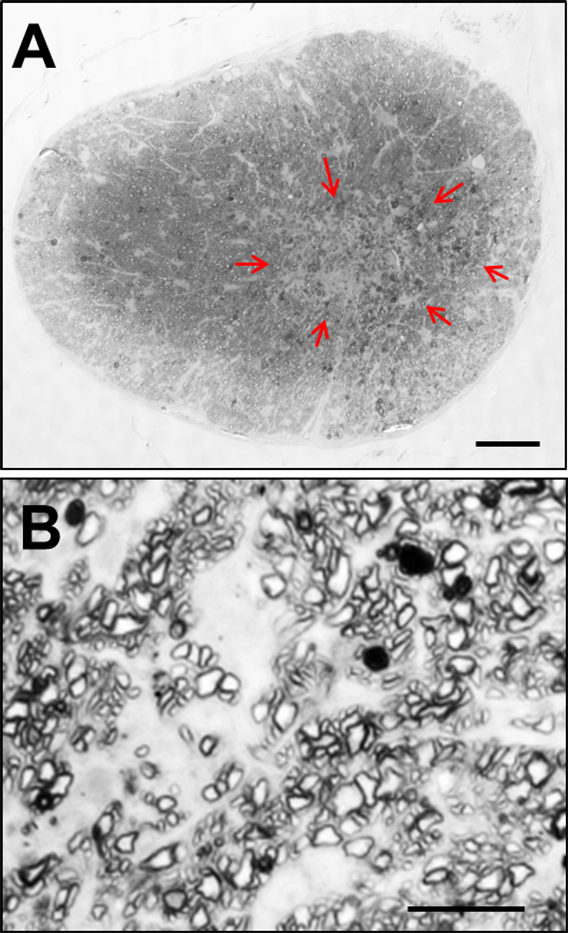

Supplement: S4 Fig — A. Optic nerve cross section stained with paraphenylenediamine (PPD) at 200x magnification. Red arrows point to a region with severe axon loss. Scale bar: 50 μm. B. Micrograph from A taken at 1,000X magnification. Scale bar: 10 μm. (TIF) [file pone.0196529.s004.tif]

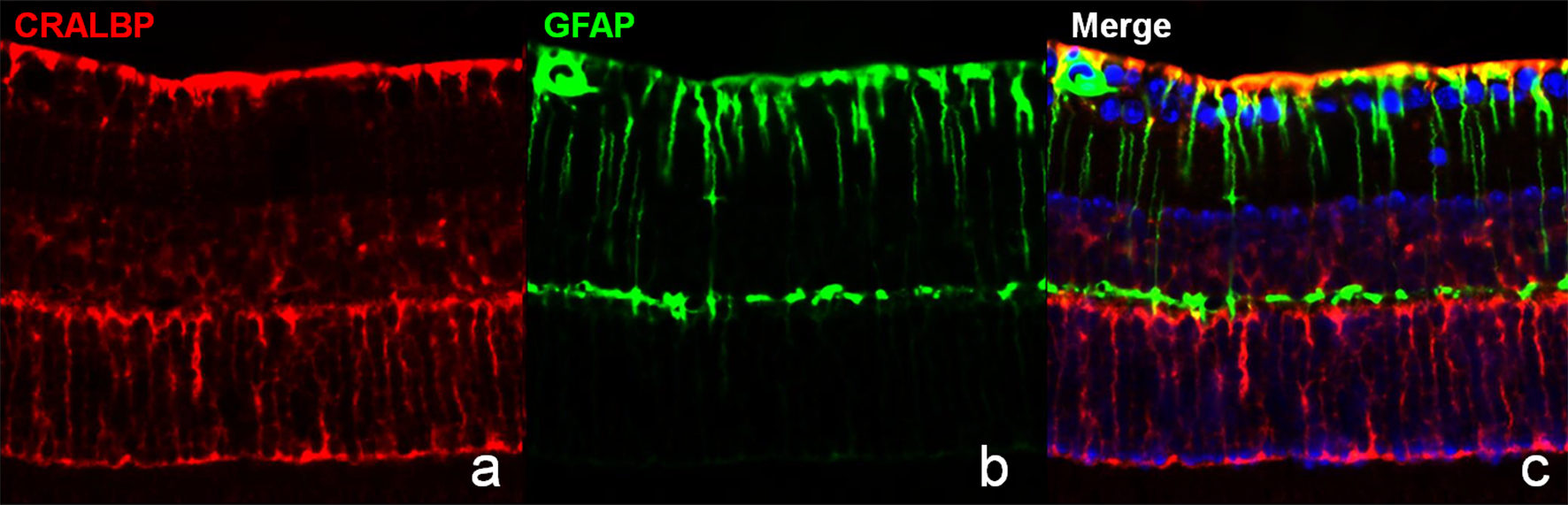

Supplement: S5 Fig — Co-immunofluorescnet staining of a hypertensive retina (Day 3) with CRALBP (a) and GFAP (b). GFAP (green) signal did not co-localize with CRALBP (red), a Müller cell marker. Blue color in (C) indicated DAPI stain. (TIF) [file pone.0196529.s005.tif]

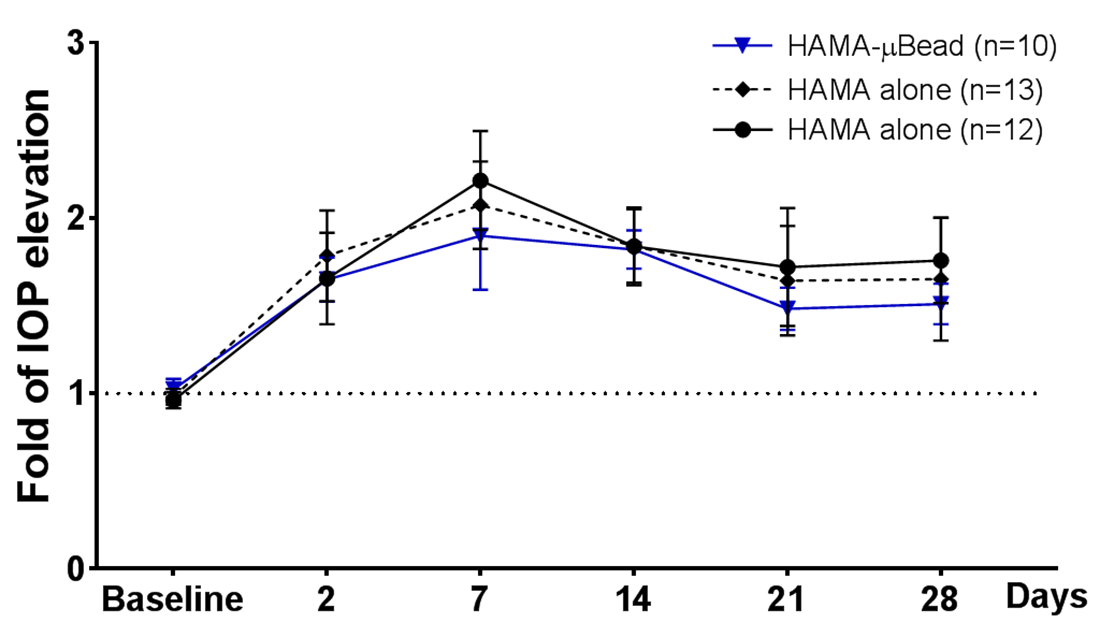

Supplement: S6 Fig — Shown here are IOP elevation curves from one additional study using 2% HAMA + microbeads, and two independent studies using 2% HAMA alone. Y axis indicates fold of IOP elevation relative to corresponding mean IOP of (PBS+UVA light) controls. n = 10–13 for control groups/study. The presence of microbeads did not impact IOP elevation. P = 0.66, Two-way ANOVA. (TIF) [file pone.0196529.s006.tif]
